# Systematic review of machine learning utilization within outpatient psychodynamic psychotherapy research

**DOI:** 10.3389/fpsyt.2023.1055868

**Published:** 2023-05-09

**Authors:** Ivo Rollmann, Nadja Gebhardt, Sophia Stahl-Toyota, Joe Simon, Molly Sutcliffe, Hans-Christoph Friederich, Christoph Nikendei

**Affiliations:** Department for General Internal Medicine and Psychosomatics, University Hospital Heidelberg, Heidelberg, Germany

**Keywords:** machine learning (ML), psychodynamic psychotherapy, outpatient therapy, review—systematic, perspectives

## Abstract

**Introduction:**

Although outpatient psychodynamic psychotherapy is effective, there has been no improvement in treatment success in recent years. One way to improve psychodynamic treatment could be the use of machine learning to design treatments tailored to the individual patient's needs. In the context of psychotherapy, machine learning refers mainly to various statistical methods, which aim to predict outcomes (e.g., drop-out) of future patients as accurately as possible. We therefore searched various literature for all studies using machine learning in outpatient psychodynamic psychotherapy research to identify current trends and objectives.

**Methods:**

For this systematic review, we applied the Preferred Reporting Items for systematic Reviews and Meta-Analyses Guidelines.

**Results:**

In total, we found four studies that used machine learning in outpatient psychodynamic psychotherapy research. Three of these studies were published between 2019 and 2021.

**Discussion:**

We conclude that machine learning has only recently made its way into outpatient psychodynamic psychotherapy research and researchers might not yet be aware of its possible uses. Therefore, we have listed a variety of perspectives on how machine learning could be used to increase treatment success of psychodynamic psychotherapies. In doing so, we hope to give new impetus to outpatient psychodynamic psychotherapy research on how to use machine learning to address previously unsolved problems.

## Introduction

Outpatient psychodynamic psychotherapy is effective in treating various psychological disorders (1–3). Further positive effects include a reduced number of sick leaves, a reduction of health care utilization, less psychiatric hospitalizations after therapy, and a reduced relapse rates for depression (4–6). A number of factors that predict successful therapy are also known, such as improving the working alliance (7, 8), therapeutic agency (7, 9, 10) or the patient's ability to perceive emotions (11, 12) which lead to a reduction in symptom burden. However, as Leichsenring et al. (13) point out, recent substantial improvements in treatment success have been scarce. The authors (13) recommend that future studies focus primarily on non-responders and drop-outs to improve available treatments. Identifying characteristics and features of non-responders and drop-outs would allow treatment to be tailored more specifically to the patients (13). However, Leichsenring et al. (13) neglect the fact that theoretical or statistical models are needed to accurately predict whether the patient's treatment will be successful or unsuccessful. We argue that such models can be developed with machine learning.

Machine learning is a field of computer science, in which the “computer” is supposed to “learn” models from data (14). “Learning” in this context means that statistical models are adapted to the data until they optimally perform a previously defined task, for example predicting drop-out rates of psychotherapy (14, 15). These statistical models can be the same methods that are used in classical statistical approaches, such as regression analyses. There is therefore no clear boundary between machine learning and classical statistical approaches (14). However, machine learning differs from classical statistical approaches in the primary way the developed models are evaluated. In classical statistical approaches, the developed models are assessed primarily with the help of statistical significance, explained variance, and many other characteristic values (16). In contrast, models in the machine learning approach are assessed primarily by how well they can perform the task they have “learned” on new data that is unknown to the often iterative model fitting process (14, 15, 17). In practice, this means that a model is “taught” by means of a first data set and then evaluated on a second data set. Machine learning approaches can further be divided into unsupervised and supervised learning (18). The primary goal of unsupervised learning is to discover relationships and structures in the data (14, 15). Commonly used statistical models for unsupervised machine learning include explanatory factor analysis, k-means clustering, and hierarchical clustering (19). While supervised learning also discovers correlations and structures in the data, the goal is to determine the value of a dependent variable as accurately as possible (14, 15). A prerequisite for this is that the dependent variable is known, both in the data set in which the model is being “taught”, and in which it is being evaluated. Commonly used statistical models for supervised machine learning include regression analysis, support vector machines, random forest, and latent discriminant analysis (19). For a more detailed description of machine learning and its own terminology, the interested reader is referred here to Dwyer et al. and Bi et al. (14, 15).

Because models developed using the machine learning approach are primarily evaluated for their ability to perform a previously defined task for new unknown data, they often perform better (assuming access to an appropriate dataset) in tasks such as predicting whether a patient's treatment will be successful, as opposed to models developed using the classical statistical approach. Machine learning thus has the potential to develop models that could lead to psychodynamic psychotherapeutic treatments being more successful. However, it is unclear whether machine learning is currently used in psychodynamic psychotherapy research. In 2019, Aafjes-van Doorn et al. (19) found 51 studies which utilized machine learning to analyse psychotherapy. Most of those studies were initial proof-of-concept studies, which either predicted the outcome of therapy, or automatically rated patient behavior for further analyses. Most of these 51 studies used transcripts of psychotherapy sessions as data and utilized supervised machine learning to answer their research questions. However, Aafjes-van Doorn et al. (19) did not differentiate between specific treatment approaches. Machine learning may have other applications in psychodynamic psychotherapy research because of the focus on the patient's unconscious. Yet, to the best of our knowledge, there seem to be no current systemic reviews about machine learning within outpatient psychodynamic psychotherapy research. Therefore, we focused our literature review on the use of machine learning in outpatient psychodynamic psychotherapy research.

## Methods

For this review, we applied the Preferred Reporting Items for systematic Reviews and Meta-Analyses (PRISMA) Guidelines.

### Search strategy and eligibility criteria

We searched the two most comprehensive databases regarding psychotherapy research, “PsycInfo” and “PubMed”. In preparation for this mini-review, we searched several databases (PsycInfo, PubMed, Heidi, Google Scholar and IEEE Xplore) for relevant literature and did not obtain any additional results beyond those from PubMed and PsycInfo. Therefore, we estimated that there would be little loss of knowledge if we omitted further databases. All 36 combinations of the terms (psychothera^*^ OR thera^*^ OR clinical assessment) AND (machine learning OR artificial intelligence OR neural network OR deep learning) AND (patient^*^ OR client^*^ OR mental health) with no limitation on publication year were searched. As there is a corpus of theoretical work comparing neural processes to artificial intelligence that considers how to use the conclusions for psychotherapy, we added the term (patient^*^ OR client^*^ OR mental health) to the search. We sought to omit such work. The two searches were conducted on 17th September 2021 and 2nd January 2023, respectively.

### Eligibility criteria

To be eligible, studies had to be original works, treat their patients with outpatient psychotherapy and use machine learning as a statistical method. Results were limited to publications in English. There were no further eligibility criteria.

### Selection process

The first and second author read all abstracts of the articles and selected the studies which appeared to meet the eligibility criteria. In a second step, the manuscripts were read and discussed among the first and second author. During this stage, studies whose psychotherapeutic treatment was not psychodynamic, or whose treatment consisted only of diagnostics, were not considered for this review. Furthermore, all studies that did not use machine learning as a statistical method were removed. Lastly, studies which treated patients with several treatment approaches, yet did not differentiate between them, were excluded, as it was impossible to attribute the results to one specific treatment approach.

### Data items

The first and second author independently retrieved the research question and the respective use of machine learning from the included studies. They also retrieved the sample size and data used for machine learning.

### Bias assessment

To assess outcome bias, we checked which characteristics the studies reported. According to Lantz (17), a study should at least report accuracy, specificity and sensitivity for an unbiased report of a machine learning model. Therefore, these aspects were taken into consideration during the selection process. Since it could be assumed that machine learning is a new field in outpatient psychodynamic psychotherapy research (19), our primary goal was to gain an overview of research conducted in this area. Therefore, a full assessment of report quality using the TRIPOD (20) criteria would have been beyond the scope of this work.

## Results

### Study selection

The initial search of both databases yielded 1,358 results, the second 6,206. In total, 3,216 were duplicate records, which were removed before screening. Of the 4,348 records screened, 4,289 were excluded, as they did not meet the eligibility criteria. Most excluded records were related to brain research and prediction of recovery processes after surgery. The second largest group of excluded records were associated with treating patients with other approaches than outpatient psychodynamic psychotherapy. In total, 59 records appeared to have met our eligibility criteria. Two records were inaccessible *via* any platform. Another 27 reports were excluded because outpatient psychodynamic psychotherapy was not a form of treatment. Nine of the 27 studies were conducted by a research group under Professor Atkins, who successfully created an automatic transcription and evaluation tool for short term psychotherapy, namely motivational interviewing (21–29). Ten of the 27 excluded studies either tried to predict the outcome of cognitive behavior therapy by using natural language processing, or tried to predict optimal therapeutic interventions with sociodemographic data (30–40). Another 16 studies excluded were reviews about machine learning and its utilization within psychotherapy, psychopharmacotherapy, and diagnostics (19, 27, 38, 41–51). Another 8 Studies were excluded because they treated patients with psychotherapy, yet did not specify which kind of treatment approach they used (22, 29, 52–57). Lastly, two studies retrieved were theoretical studies (58, 59). To summarize, only 4 out of 4,348 records screened were eligible for our review.

### Study characteristics and results

An overview of the four studies that were deemed eligible, as well as the utilized machine learning methods and their bias assessment, can be seen in Tables 1, 2. Two of the four studies were single-dyad studies. Villmann et al. (63) examined the possible use of artificial neural networks to investigate psycho-physiological parameters derived during the therapy sessions. The authors measured five physiological parameters across 37 therapy sessions for both the patient and therapist and transcribed all sessions. The machine learning model applied was a growing-self-organizing map to combine the psychophysiological data into emotional entropy. Emotional entropy can be understood as emotional variability or emotional energy. The session transcripts were processed with the Mergenthaler Cycle Model (64), which groups the words in the transcripts into four topics: relaxing, experiencing, reflecting and connecting. The authors then compared the emotional entropy with the Mergenthaler Cycle topics. In doing so, they found a cyclic process (64). The patient experiences an interpersonal conflict which increases emotional entropy. The conflict is then reflected upon, and the patient connects the interpersonal conflict with an inner conflict. This connection unleashes emotional energy, which enables a structural change within the patient. Afterwards, a period of relaxation and stabilization follows. Villmann et al. (63) described their proof-of-concept study as an initial first step, which should be verified in future studies.

**Table 1:** Study characteristics.

**First Author**	**Study Design**	**Sample**	**Research question**	**Data used**
Atzil-Slonim et al. (60)	Longitudinal study, no RCT, exploratoty study	873 therapy sessions from 58 patients and 52 therapists	Prediction of symptom reduction and social functioning based on topics spoken about in therapy	Transcript of therapy sessions, Symptom Checklist, Outcome Rating Scale
Halfon et al. (61)	Longitudinal study, no RCT, exploratory study	148 therapy sessions from 53 children and 24 therapists	Prediction of a child's affect expressions based on video or transcription of therapy sessions	Video and Transcript of sessions, Affect expression scale of the Children's Play Therapy Instrument
Laskoski et al. (62)	Longitudinal study, proof-of-concept	120 therapy sessions from 1 patient and 1 therapist	Prediction of Patient distress based on therapist behavior and interventions	Psychotherapy Process Q-set (requires video of sessions), Outcome questionnaire
Villmann et al. (63)	Longitudinal study, proof-of-concept	37 therapy sessions from 1 patient and 1 therapist	Studying behavior of psychophysiological variables within therapy	Heart rate, respiratory frequency, muscular tension, skin conductance response, skin conductance level, transcript of sessions

**Table 2:** Utilized machine learning models.

**First author**	**Machine learning models**	**Type of machine learning**	**Application in paper**	**Bias assessment**
Atzil-Slonim et al. (60)	Latent dirichlet allocation	Unsupervised	Extracting topics discussed in psychotherapy session	Potentially biased. Authors only report accuracy.
	Sparse multinomial logistic regression	Supervised	Predicting Patient Outcome based on topics discussed in therapy session	
Halfon et al. (61)	Dictionary approach	Unsupervised	Generating affect scores from session transcripts, based on existing corpora	Not applicable. Authors correlate results with human raters. Instead of assessing accuracy, specificity and sensitivity of model.
	Deep neural network	Both	To generate valence and arousal scores from therapy videos (pre-trained on affect net database)	
	Support vector machine	Supervised	Predicting the affect of children with on the generated affect scores	
	Extreme learning machine	Supervised	Predicting the affect of children with the generated affect scores	
Laskoski et al. (62)	Random forest algorithm	Supervised	Predicting patient distress with coded therapist behavior and interventions	Potentially biased. Authors only report values from best model.
Villmann et al. (63)	Dictionary approach	Unsupervised	Grouping Words of session transcripts into Mergenthaler Cycle topics	Potentially biased. Authors report neither accuracy, sensitivity nor specificity.
	Growing Self-Organizing Map	Unsupervised	Creating a lower dimensional description of psychophysiological data (comparable to a non-linear Principal component analysis)	

The second single-dyad study was done by Laskoski et al. (62). They used a random-forest model to predict patient distress based on coded interventions from a videotaped psychoanalysis, consisting of 120 sessions. Trained judges rated the psychotherapist's interventions with the Psychotherapy Process Q-Set (65). The patient answered the Outcome Questionnaire after each session (66). The random-forest model had an AUC of 0.725, sensitivity of 79%, specificity of 79%, and accuracy of 70.5 % in predicting patient stress after therapy sessions. Additionally, the authors calculated the variable importance of the predictors and found standard techniques of psychodynamic therapy, e.g., drawing the patient's attention to unconscious content or linking the patient's feelings to past situations, to be the most important factors in reducing patient distress.

The third study was conducted by Atzil-Slonim et al. (60). They used Latent Dirichlet allocation to extract various topics from session transcripts. Then, a sparse multinomial logistic regression was used to predict the social functioning and symptom distress after each therapy session based on the topics discussed. Social functioning and symptom distress of the patient were measured with the Outcome Rating Scale and Symptom-Checklist (67, 68). In total, they analyzed 873 therapy sessions deriving from 58 patients and 52 therapists. Results showed that an increase in positive topics was positively correlated with high social functioning and associated with a decrease in symptoms distress. Conversely, an increase of negatively connotated topics correlated with an increase of symptom distress. Accuracy of the final model was at 75.6 % with regard to predicting social functioning.

Halfon et al. (61) tried to predict four basic emotions (joy, anger, sadness and anxiety) of children within a psychodynamic play therapy. Their sample consisted of at least two randomly drawn videotaped sessions per therapy. In total, 148 videotaped sessions of 53 children and 24 psychotherapists were selected. Emotional expressions of children were coded by trained judges using the affect expression items of the Children's Play Therapy Instrument (69). The videos were transcribed separately. Afterwards, the authors trained several supervised machine learning models to predict the affect expressions of children based on the transcript or the video. Overall, a fusion strategy, which combined text analysis and facial recognition to predict affect expressions, achieved the best results. Still, affect expression predictions of the final model correlated on average *r* = 0.30 with the ratings of trained judges. Halfon et al. (61) concluded that the “automatic affect analysis is promising, however, needs further development.”

### Synthesis of results

Our review identified four studies that utilized machine learning within outpatient psychodynamic psychotherapy research. All four studies are proof-of-concept studies. Furthermore, none of the four studies reported their results without bias. All four studies differed in the type of data they used for their machine learning models and their study aims. All studies trained their machine learning models with data from completed outpatient psychodynamic psychotherapies only. Lastly, three of the four studies were done within the last 5 years.

## Discussion

Some authors call for psychodynamic psychotherapies to be tailored to patients as much as possible in order to be more successful (13). However, this requires models that allow an accurate prediction of whether the therapy will be successful or unsuccessful. We argue that models developed using the machine learning approach are particularly well suited for this purpose. Within machine learning, models are evaluated primarily for their ability to perform a predetermined task on new data (14, 15). However, since it was unknown how widely machine learning is represented in outpatient psychodynamic psychotherapy research, we conducted a review. Our systematic review identified four proof-of-concept studies that utilized machine learning. Three studies were published between 2019 and 2021 and two studies had a single-dyad sample. All four studies utilized machine learning to evaluate completed outpatient psychodynamic psychotherapies. It seems that machine learning has only recently entered outpatient psychodynamic psychotherapy research. However, this could also mean that researchers are not yet aware of what machine learning can be used for in psychodynamic psychotherapy research. We therefore want to present perspectives and ideas that can be used for future psychodynamic psychotherapy studies. We would then like to highlight a possible risk that might occur when using machine learning in psychotherapy.

To make psychotherapies more successful, Leichsenring et al. (13) suggest that therapy should be tailored to the needs of patients with high non-response and drop-out probability. A possible implementation of this idea would be to predict therapy success or drop-out at the beginning of the therapy and to include these predictions in the therapy planning. Some studies within cognitive behavioral therapy research attempted to implement this (42, 53, 70, 71). A commonality of these studies is that socio-demographic data was collected before the start of psychotherapy and used to predict psychotherapy success with the help of machine learning models. Psychotherapy success was operationalised differently, either as symptom improvement, drop-out or improvement in quality of life.

Another implementation of the idea of tailoring therapy to patients' needs could include feedback to the therapists. De Jong et al. (72) were able to show that feedback to the therapists reduces the drop-out probability of patients by 20% and leads to stronger symptom improvement. Machine learning can be used in this context to develop models that automatically evaluate audio transcripts of psychotherapy sessions and provide feedback to the therapist. The therapist would then be able to get timely feedback about possible pathological developments and could intervene accordingly. A research group led by Professor Atkins is currently attempting to implement this idea (21, 22, 25, 26, 28). Currently, they are only successful in doing this for the very standardized Motivational Interviewing (21). However, it seems possible to build on the work of this group and provide therapists with feedback on variables that are relevant to psychodynamic therapy, such as the agency, working alliance, and the patient's structural integration of personality (7, 8, 11).

A third way to tailor therapy to patients' needs is to predict the fit between therapist and patient. Delgadillo et al. (33) found that there are differences between therapists in the effectiveness with which they treat individual patient groups. Their final machine learning model identified 17 classes of patient-to-therapist matches, which vary greatly in their effectiveness. Building on this idea of Delgadillo et al. (33), it would be conceivable to develop a machine learning models that can predict which therapist has the highest probability of achieving a successful therapy with a patient.

On the other hand, the use of machine learning in psychotherapy research should be carefully considered. As the previous ideas illustrate, models developed with machine learning have the ability to automate many processes, such as feedback to and allocation of patients to therapists. This poses the risk that such models could become an unreflective and potentially discriminatory standard (26, 73). In other words, minorities and vulnerable groups are disadvantaged, for instance, by being denied psychotherapeutic treatment because the model predicts that treatment will be unsuccessful. In this context, Hirsch et al. (26) examined how well their model, which gave feedback on Motivational Interviewing, was accepted by therapists. They found that novices in particular tended to accept the feedback without reflection. Therefore, Besse et al. (73) warned that this can also systematically create discrimination, especially if the model was not developed on the basis of theoretical considerations and representative data (73, 74). The use of machine learning in psychotherapy research should therefore be embedded in existing theories.

## Limitations

Several limitations of the presented work must be mentioned. Some studies which utilized machine learning as a method within outpatient psychodynamic psychotherapy research may not have been considered in our review, as we had rather strict criteria for inclusion. Although Zilcha-Mano et al. (56) treated some patients with psychodynamic psychotherapy, it was excluded in our review, as they did not differentiate their results between treatment approaches. Furthermore, we only included studies which explicitly mentioned, in their abstracts, that they used machine learning, deep learning or a form of artificial intelligence. It is conceivable that articles referring to their methodology with the name of statistical model, instead of machine learning, were not included. Furthermore, articles that did not mention their methodology within the abstract, although they used machine learning, may also have been disregarded. As we only found four studies within outpatient psychodynamic psychotherapy research that used machine learning, our review summarizes the first attempts at adopting machine learning into this field of research. Therefore, various limitations mentioned by Aafjes-van Doorn et al. (19) also apply to this review. As these are among the first studies in this area, they should be interpreted cautiously, namely as proof-of-concepts studies, so that the significance of their results is not overestimated. Therefore, we also refrained from assessing the quality of the studies using TRIPOD criteria (20). Thus, we recommend that future reviews in this field use the TRIPOD criteria (20) to assess the quality of studies.

## Conclusion

Although much research has been done on psychodynamic psychotherapy, the treatment success of this therapy method has not improved. We argue that machine learning is a way to develop models that detect non-responders and patients with high drop-out probability early and enable intervention. However, since it was unknown how widespread machine learning is in outpatient psychodynamic psychotherapy research, we felt it necessary to conduct a review of current research. We found four studies, three of which were carried out between 2019 and 2021. Thus, machine learning seems to have entered this field of research only recently and researchers might not yet be aware of its possible uses. We have therefore outlined some possibilities, ideas, and perspectives on how machine learning can be used to improve the success of psychodynamic psychotherapies. Thus, we hope to give new impetus to outpatient psychodynamic psychotherapy research on how to use machine learning to address previously unsolved problems.

## Author contributions

IR planned the study, conducted the literature search, and wrote the manuscript. NG was responsible with IR for selecting the studies to include into the review. SS-T used her expertise to correct the parts about machine learning in the manuscript. JS was an advisor about machine learning. MS was substantially aided in improving the accuracy of the results and discussion. H-CF and CN were the supervisors for this study. All authors contributed to the article and approved the submitted version.
